# ‘Making the most of together time’: development of a Health Visitor–led intervention to support children’s early language and communication development at the 2–2½-year-old review

**DOI:** 10.1186/s40814-022-00978-5

**Published:** 2022-02-08

**Authors:** Cristina McKean, Rose Watson, Jenna Charlton, Sue Roulstone, Caitlin Holme, Victoria Gilroy, James Law

**Affiliations:** 1 Newcastle University, Newcastle upon Tyne, UK; 2 Bristol Speech and Language Therapy Research Unit, North Bristol NHS Trust, Southmead Hospital, Bristol, UK; 3 University of West of England, Bristol, UK; 4 Institute of Health Visiting, London, UK

**Keywords:** Language development, Young children, Intervention development, COM-B model, Implementation science, Tailored intervention, Preventative intervention, Shared decision-making, Intervention equity

## Abstract

**Background:**

Early interventions to support young children’s language development through responsive parent–child interaction have proven efficacy but are not currently delivered universally. A potential universal delivery platform is the Health Visitor (HV)–led 2–2½-year-old review in England’s Healthy Child Programme. It is unclear if it is feasible to offer such interventions through this platform. We report an intervention development process, including extensive stakeholder consultation and co-design which aimed to develop an acceptable, feasible and equitable early language intervention for delivery in this context.

**Methods:**

The study involved five phases including 13 stakeholder co-design workshops with 7 parents and 39 practitioners (HVs, early years practitioners and speech and language therapists): (1) Identification of existing intervention evidence, (2) qualitative review of intervention studies extracting candidate target behaviours for intervention and intervention techniques, (3) co-design workshops with parents and practitioners examining acceptability, barriers and enablers to those behaviours and techniques (particular attention was paid to diverse family circumstances and the range of barriers which might exist), (4) findings were analysed using COM-B and theoretical domains frameworks and a prototype intervention model designed, and (5) co-design workshops iteratively refined the proposed model.

**Results:**

Practitioners were committed to offering language intervention at the 2–2½-year-old review but were not sure precisely how to do so. Parents/caregivers wanted to be proactive and to have agency in supporting their own children and to do this as soon as possible. For equitable intervention, it must be *proportionate*, with higher ‘intensity’ for higher levels of disadvantage, and *tailored,* offering differing approaches considering the specific barriers and enablers, assets and challenges in each family. The importance and potential fragility of alliances between parent/caregiver and practitioner were identified as key, and so, strategies to engender successful collaborative partnership are also embedded in intervention design.

**Conclusion:**

It is possible to develop a universal intervention which parents and practitioners judge would be acceptable, feasible and equitable for use at the 2–2½-year review to promote children’s language development. The result is one of the most explicitly developed universal interventions to promote children’s language development. Further development and piloting is required to develop materials to support successful widespread implementation.

**Supplementary Information:**

The online version contains supplementary material available at 10.1186/s40814-022-00978-5.

## Key messages regarding feasibility

1. What uncertainties existed regarding the feasibility?
  Early interventions to support young children’s language development through responsive parent–child interaction have proven efficacy but are not currently delivered universally.
  A potential universal delivery platform is the Health Visitor (HV)–led 2–2½-year-old review in England’s Healthy Child Programme.
  It is unclear if it is feasible to offer such interventions through this platform.
2. What are the key feasibility findings?
  It is possible to develop a universal intervention for use at the 2–2½-year review to promote children’s language development which practitioners and parents consider would be acceptable, feasible and equitable.
  For equitable intervention, it must be *proportionate*, with higher ‘intensity’ for higher levels of disadvantage, and *tailored,* offering differing approaches considering the specific barriers and enablers, assets and challenges in each family.
  The importance and potential fragility of alliances between parent/caregiver and practitioner were identified as key, and so, strategies to engender successful collaborative partnership are essential for success and must also be embedded in intervention design.
3. What are the implications of the feasibility findings for the design of the main study?
  The intervention devised provides a protocol for delivery which participants felt would be acceptable, feasible and equitable; however, it is untested in practice.
  Further work is also needed for ELIM-I to be accessible to families from a range of linguistic and cultural backgrounds.
  Further work is required to develop and pilot a manualised programme with standardised intervention resources and guidance for local implementation and policy development.

## Background

The early years of a child’s life lay the foundation for their health, education and wellbeing across the life course [1, 2]. A child’s language development in these early years is a key component of that foundation and is now widely recognised as a crucial indicator of an individual’s ‘life chances’ [3]. Children who enter school (aged 5–6 years) with language difficulties are at risk of poorer long-term outcomes with respect to adolescent educational attainment and social–emotional wellbeing [4], adult literacy, mental health, employment [5, 6], health literacy [7], social anxiety and isolation [8]. Given that prevalence estimates suggest that, on average, every primary school classroom in England contains two children with significant language difficulties [9], reaching up to 40% in the most disadvantaged communities [10], this is a substantial challenge for educational, social and health services.

Many social and educational policies around the world make robust early language development a key objective [11–13]. A great deal is known about the aspects of a child’s early learning environment which can be harnessed to promote positive language outcomes in the pre-school period due to several systematic reviews and efficacy and epidemiological studies [14–22]. Despite this, the development of an intervention, which can be delivered universally, affordably and effectively for children under the age of 3 years, has remained elusive. Recently, the case has been made that early language and communication needs should be tackled through public health preventive models of intervention [23]. In the UK, the Healthy Child Programme provides support from 0 to 19 years of age and is led by Public Health England (PHE) aiming to ‘improve the health of babies, children and their families to enable a happy healthy childhood and provide the foundations of good health into adult life’ [24–26]. In the 0–5-year period, this programme of work is led by Health Visiting Teams. HVs are specialist nurses, and their teams often include trained early years educators. The teams are separate from but closely linked to family doctors and paediatric services and provide families with a programme of screening, immunisation and health and development reviews, supplemented by advice around health, wellbeing and parenting. In 2018, PHE identified early language development as one of six ‘high impact’ areas where HV services can make the greatest difference [27]. Whilst such ambitions and their rationale are clearly stated in policy, precisely *how* to ensure all children are supported to achieve positive language outcomes is less well specified. Furthermore, whilst the HV 2–2½-year-old review has been the focus of timely identification of speech, language and communication needs (SLCN) using the Ages and Stages Questionnaire (ASQ-3) nationally mandated pre-assessment tool [28], the mode, content and delivery of this contact is variable, and interventions commonly lack conceptual and practical detail. In recognition of this, the Department for Education (DfE) commissioned a programme of training and research to develop a national approach to support the development of children’s early language and led by PHE [29]. This research strand developed a novel tool for HV teams to identify children at risk of poor language development and a linked intervention: the Early Language Identification Measure and Intervention (ELIM-I) [30, 31]. Here, we focus on the development of the *intervention* component of the ELIM-I, whilst a description of the development of the identification measure is reported elsewhere [32].

This paper outlines the development of a universal intervention to offer to families at the HV 2–2½-year-old review to promote robust language development for all children. Our aim was to develop an intervention which aligns with the aims, principles and structure of the modernised HCP [33]. That is, an approach with ‘universal reach and a personalised response’ to be led by HV teams in England, and which focusses, in the first instance, on the universal 2–2½-year-old review and draws on the wider children’s workforce, as necessary. Following guidance on the development of complex and public health interventions, we sought to develop an intervention which is acceptable, equitable, practicable, can be delivered at scale, and which is based on current best evidence and underpinned by relevant theory [34–36].

As with any public health intervention, there is a risk that universal approaches can inadvertently widen rather than narrow inequalities if the necessary attention is not paid to structural factors which influence a family’s ability to engage in a given health-promoting behaviour [37]. There is evidence to suggest this is a real risk for early language interventions [38, 39]. An alternative is to apply ‘proportionate universalism’ where intensity of action is proportionate to the level of disadvantage [40]. However, *intensity* is not the only characteristic which can and should be tailored to the individual circumstances of a family. Much of the existing evidence regarding pre-school language interventions focusses on building capacity in parents/caregivers: their knowledge and skills as to how to create a language enriching environment for their child. Insufficient attention has been paid to other factors associated with structural inequalities such as families’ opportunities and resources as well as affective factors such as their optimism and belief about their capabilities [41, 42]. For an equitable intervention to be designed, we must not only create a *proportionate* model (i.e. with higher ‘intensity’ for higher levels of disadvantage need [40]) but also a *tailored* one, offering differing approaches considering the specific barriers and enablers, assets and challenges in each family [43].

In conclusion, the aim was to develop an intervention that is

- Acceptable, practicable and can be delivered at scale;
- Based on current best evidence and underpinned by relevant theory;
- Proportionate to the assets and challenges of individual families;
- Tailored to the barriers and enablers present for individual families; and
- Well specified in its methods to enable fidelity in delivery.

## Overarching methodology

The Medical Research Council’s guidance for the development and evaluation of complex interventions emphasises the importance of rigorous intervention development [34]. However, it is only relatively recently that detailed, systematic and replicable methods for this first phase of intervention research have been specified [41, 44]. Our methods align with the most recent guidance by O’Caithan and colleagues published in 2019 [35], and are an adaptation of those described by O’Brien et al. [44]. This iterative and sequential method is designed to enable the integration of published scientific evidence, expert knowledge and experience and detailed consideration of stakeholder knowledge and views. We remained open to change, and processes were developed and adapted as necessary in response to outcomes at each stage. We made use of the expertise of the research team at several stages to challenge, develop and contextualise intervention development. The team comprised researchers with backgrounds in Speech and Language Therapy, General Practice, Health Visiting (practice and policy), Psychology, Medical Sociology and Linguistics. In addition, reflections from a parallel study regarding the acceptability of the ELIM/developmental review were used to challenge interpretations/analyses (Holme C, et al. Parental experiences and perspectives of the 2–2½ year developmental review process for identifying speech, language and communication needs. Under review). Furthermore, we report the intervention development following recognised reporting guidance (GUIDED) [45].

### Theoretical perspective

We drew on existing theory in several ways. First, with respect to child language development, we were informed by socio-cognitive theories [46–48] which emphasise the importance of responsive interactions with caregivers for robust language development. A number of infant socio-cognitive skills are also crucial to early language development: the ability to share attention with adults, understand their communicative intentions and take turns in conversations [49]. Language is learned best in responsive social interactions between caregiver and infant where the language used by the adult is contingent on the child’s attention and where the child is deploying these socio-cognitive abilities to infer meaning and maintain the interaction [19, 21, 22, 49]. Importantly, caregiver-responsive contingent interactions also facilitate the development of these socio-cognitive abilities and so are critical to robust language and communication development from the very earliest days of a child’s life [50]. Second, we planned to apply Behaviour Change Theory to the intervention development drawing on the Behaviour Change Wheel [41] and the Theoretical Domains Framework (TDF) [42]. Third, the Theoretical Framework of Acceptability (TFA) [51] informed the development of stakeholder co-design workshop materials.

As data were collected and analysed, it became clear that consideration of the socio-relational aspects of the intervention was essential and must inform intervention design if an effective and acceptable approach was to be developed. Additional theory relating to principles of shared decision-making, therapeutic alliance, trust and engagement was therefore also consulted [36, 52–59].

When attending to future implementation, normalisation process theory (NPT) was also considered [60]. Embedding health care innovations into routine practice is not straightforward and requires explicit planning. NPT suggests that four kinds of work need to occur for an innovation to become ‘normalised’ practice: coherence work (or sense-making), participation work (or engagement), enacting work (action to enable the intervention to happen) and appraisal work (reflection and monitoring of the benefits and costs) [61]. When designing the intervention, where HV teams were on the ‘journey’ towards normalisation of support for children’s language development was considered to ensure the approach devised takes the necessary next steps. Future implementation was also considered through the lens of acceptability and considering APEASE criteria (Affordability Practicability, Effectiveness and cost-effectiveness, Acceptability, Safety and Equity) throughout the stages of development [41, 51].

### Ethics

All relevant details about the project were submitted to the West Midlands–Black Country NHS Research Ethics Committee (REC), and a favourable ethical opinion was received on the 7th of May 2019 (REC reference 19/WM/0114 project # 261205). Research and development (R&D) management approvals were then received from the five sites involved in the study. Participants gave fully informed consent before each workshop/data collection episode.

### Design

**Fig. 1:**
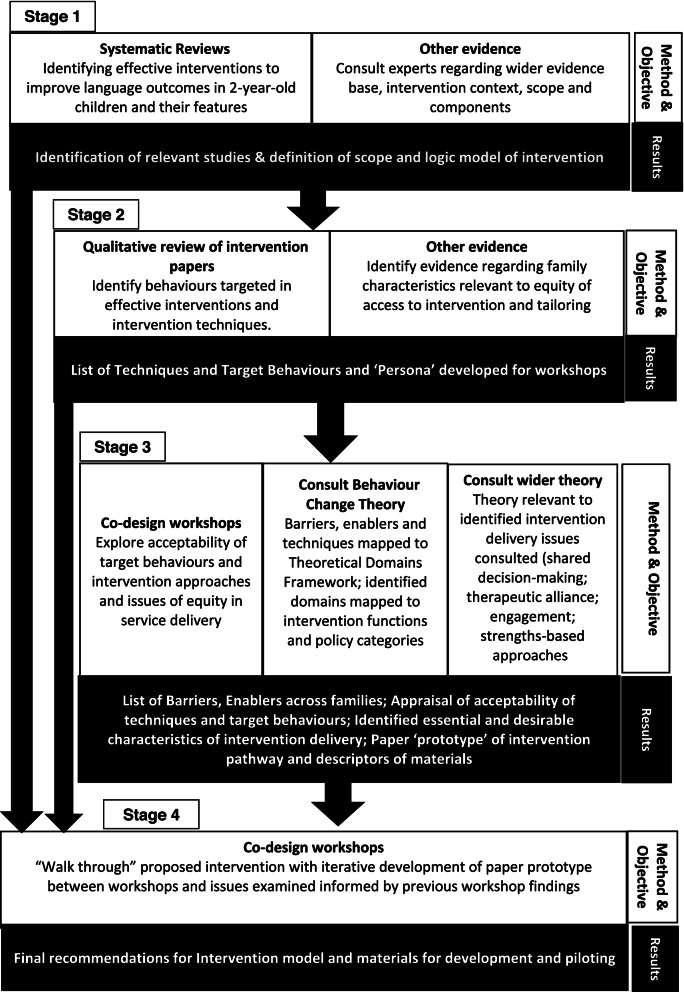
Overview of the intervention stages, outputs and their linkage

An iterative design process was followed through which evidence was gathered and appraised, relevant theory identified and applied, and intervention models and materials generated, tested and analysed. There were four stages, each stage providing results which then formed the basis of the design of the next phase (Fig. 1). Stages 3 and 4 comprised co-design workshops with parents and practitioners. A total of 13 stakeholder co-design workshops were completed: seven in Stage 3 and six in Stage 4. Members of the research team reflected on and discussed processes and outputs over the course of the study. A number of Public Patient Involvement (PPI) workshops also contributed to knowledge of the context of the interventions.

### Sites

PHE led a selection process to identify 5 sites to host the ELIM-I study based on prevalence of speech, language and communication needs (SLCN) (as indicated by school readiness), prevalence of risk factors associated with SLCN (including free school meal eligibility as a proxy for socio-economic status and English as an additional language) and the availability of site data. The sites included a mix of urban, rural, northern and southern geographies and a range of service delivery models [32]. Table 1 presents a summary of the demographic detail for the local authority areas of the 5 sites drawing on data from the Office of National Statistics [62, 63].

**Table 1:** Summary on demographic detail for the local authority areas of the 5 sites

Site	Local authority income deprivation decile^a^	English region^a^	Urban/rural classification^a^	Population proportion by ethnic group White British/ BAME^b,c^	
1	1	North East	Urban with city and town	83%	17%
2	2	East Midlands	Urban with city and town	78%	22%
3	7	South West	Largely rural	92%	8%
4	2	North	Urban with city and town	89%	11%
5	1	South East	Largely rural	15%	85%

^a^Data source: https://www.ons.gov.uk/visualisations/dvc1371/#/E09000023

^b^Data source: https://www.ons.gov.uk/peoplepopulationandcommunity/populationandmigration/populationestimates/datasets/populationcharacteristicsresearchtables

^c^BAME Black Asian and Minority Ethnic groups including White non-British groups

### Participants

Study contacts at each site provided meeting facilities and acted as gatekeepers to participant recruitment. For practitioners, study contacts were asked to invite members of the HV team (HVs and community nursery nurses (CNN)) and relevant members of the Speech and Language Therapy (SLT) team. For parents/caregivers, they were asked to invite parents of children aged 3–6 years currently receiving support for their SLCN (Table 2). This was to allow us to engage with the experiences of families with recent experience of the pathway from identification to receipt of support. Seven parents were involved across the workshops. Two parents attended two co-design workshops, and five attended one. Parents were given shopping vouchers as a token of appreciation for their time, and their travel expenses were reimbursed.

**Table 2:** Stakeholder workshops and participant characteristics

Stage	Site	Group	Sample size	Professional groups	Code
3	1	Parents/caregivers	2		P-C-WS1
		Practitioners	3	2 HVs, 1 student HV	Prac-WS1
		Practitioners	2	1 HV, 1 student HV	Prac-WS2
	2	Parents/caregivers	2		P-C-WS2
		Practitioners	5	3 HV, 2 SLTs	Prac-WS3
	3	Parents/caregivers	2		P-C-WS3
		Practitioners	6	2 HVs, 2 SLTs, 2 SLTAs	Prac-WS4
4	1	Parents/caregivers	1		P-C-WS4
		Practitioners	10	5 HVs, 2 CNNs, 2 SLTs	Prac-WS5
	3	Parents/caregivers	2		P-C-WS5
		Practitioners	8	4 CNNs, 2 SLTs, 2 SLTAs	Prac-WS6
	4	Practitioners	3	3 HVs	Prac-WS7
	5	Practitioners	9	5 HVs, 1 family nurse, 2 CNNs, 1 student nurse	Prac-WS8

Stage 5 parent/caregiver workshops in Site 5 was cancelled due to COVID-19 restrictions on travel and were offered in Sites 2 and 4 but not attended by any parents; all CNNs were part of HV teams. Codes are used when reporting quotes from workshops in the Results section

Key: *HV* Health visitor, *CNN* Community nursery nurse, *SLT* Speech and language therapist, *SLTA* Speech and language therapy assistant, *Prac* Practitioner, *P-C* Parent/caregiver, *WS* Workshop

Thirty-nine different practitioners were involved across the workshops. A range of practitioner roles were represented with the substantial majority being HVs or community nursery nurses working within the HV team. This allowed for issues of acceptability, practicability, implementation and equity to be explored. SLTs were also represented to draw on their knowledge of local SLCN pathways and of successful language intervention models and techniques. A total of seven different parents/caregivers participated, one of whom spoke English as an additional language, and 36 different practitioners (18 HVs, 6 community nursery nurses, 2 student HVs, 1 family nurse, 1 student nurse, 6 speech and language therapists, 2 speech and language therapy assistants). Parent/caregiver participant recruitment was affected by the government restrictions associated with COVID-19. Attendance at offered workshops in Sites 4 and 2 were probably affected by the growing anxiety at that time, and a final planned workshop in Site 5 with twelve families recruited had to be cancelled due to travel restrictions immediately prior to the first UK lockdown.

## Methods and results

Due to the iterative and interconnected nature of the study design, the following presents the methods and results for the four stages in turn. Figure 1 summarises the methods, objectives and results of each stage and their linkage.

### Stage 1 methods

To identify evidence-based interventions in a parsimonious fashion, the starting point for Stage 1 was previous systematic and scoping reviews [15, 16, 64–67]. This ensured a level of quality assurance and relevance without the need for full systematic reviewing of the intervention literature. The knowledge and expertise of the team was utilised within a workshop to review and analyse identified intervention studies to determine their quality and relevance to the HV check (see Supplementary materials 1 for additional detail) and to make explicit key relevant components of the interventions.

### Stage 1 results

**Fig. 2:**
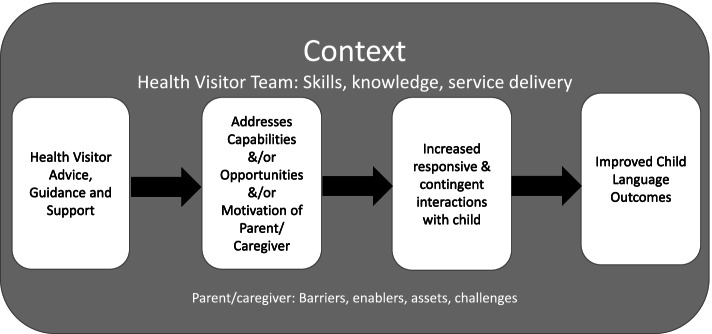
Simple Logic Model to guide intervention development

A final set of 16 papers was identified detailing effective interventions of relevance to the HV assessment (see Supplementary materials 1). A simple preliminary logic model of the intervention was designed based on initial review of the interventions and workshop discussions (Fig. 2).

### Stage 2 methods

The intervention papers identified in Stage 1 were examined and the target behaviours of the effective interventions and the intervention techniques extracted.

‘Personae’ were developed for use in practitioner workshops at Stage 3 to explore how any intervention would need to be tailored for different families to ensure equity and proportionality [68]. These personae described families with whom the practitioner might work which vary according to characteristics that may affect a family’s ability to engage with an intervention and/or the target health-promoting behaviour. Their use in our data collection methods aimed to make explicit expert practitioner knowledge and clinical decision-making, which can often be tacit or implicit [69]. To develop persona in an objective, empirically based manner and avoid the danger of pejorative or reductive stereotypes, we searched for epidemiological studies which consider how potential barriers and enablers to positive language outcomes cluster within families. The resulting personae were based on work by Christensen and colleagues using latent class methods, which identified, six distinct clusters of family risks associated with differing vocabulary growth trajectories in a representative sample of 4000 Australian children [70]. ‘Pen portraits’ of families representing each of the risk clusters described by Christenson et al. were developed using gender neutral names from a range of cultural heritages. Personae were developed only for use with practitioners; parents/caregivers were asked instead to reflect on their own experiences to maintain the validity of the insights gained as emerging from their own lived experience rather than any assumptions or generalisations about other family circumstances.

Workshop materials were designed using the findings above and drawing on behaviour change and acceptability theoretical frameworks [41, 51]. They aimed to elicit parent/caregiver and practitioner opinions regarding the acceptability of intervention target behaviours and techniques, and barriers and enablers for families with differing assets and challenges.

### Stage 2 results

Table 3 lists the target behaviours and intervention techniques extracted from the list of intervention papers. Multiple goals were targeted within complex interventions falling broadly into three categories: responsive contingent interaction, shared book reading and focussed stimulation. Several intervention techniques were used including video feedback; multiple techniques were identified including video coaching, diary completion, environmental prompts (e.g. fridge magnet reminders), etc.. The persona ‘pen portraits’ are presented in Table 4. Workshop materials are available on request from the authors.

**Table 3:** Workshop descriptors of the behaviours targeted and intervention techniques used in the research literature

Intervention type	Behaviour(s) targeted by intervention	Intervention techniques
Shared/dialogic book-reading	Share an age-appropriate book with your child for 10–15 min per day for 5 or more times per week. While sharing the book 1. Ask open questions like ‘where, who what……..’ 2. Avoid questions where your child might answer ‘yes’ or ‘no’ or just point 3. When your child answers, follow up with another question 4. Follow the child’s interests in the book 5. Praise them for good answers and ideas 6. Expand what the child says—so if they say ‘ball’, you say ‘yes, a big ball’	• You are given gifts of age-appropriate books • You watch the Health Visitor show you how to share the book using the recommended behaviours • While you share the book using the recommended behaviours, you are videoed and then you and the Health Visitor look at the video together and see what you might change • You attend a group at a community centre or library to work with other parents to learn the recommended techniques for book sharing—you watch videos of other parents sharing books with their child and discuss what they might change • You are phoned weekly to see how you are doing • You are given a leaflet describing the techniques to use when sharing books with your child • You are asked to keep a diary of when, where and for how long you share a book with your child • The Health Visitor explains to you how shared book reading benefits your child
Responsive/contingent interaction	During every day activities and routines, you are asked to communicate with your child in a ‘responsive’ way by 1. Following the child’s lead and interests 2. Pausing and waiting to see what they are interested in 3. Listening, watching and responding to their communication—these may be words, points, sounds or movements 4. Describe what your child is doing 5. If they say anything, imitate and expand what they have said—so if the say ‘shoe’, say ‘yes, that’s Molly’s shoe’ 6. Have fun, and show them you are having fun	• You are given gifts of age-appropriate toys which will help you to follow their lead. You watch the Health Visitor show you how to play with your child using the recommended ‘responsive communication’ behaviours • While you play with your child using the recommended ‘responsive communication’ behaviours, you are videoed and then you and the Health Visitor look at the video together and see what you might change • You attend a group at a community centre or library to work with other parents to learn the recommended techniques for ‘responsive communication’; you watch videos of other parents playing with their child and discuss what they might change • You are phoned weekly to see how you are doing • You are given a leaflet describing the responsive communication behaviours to use when playing or in everyday activities with your child • You are asked to set aside 15 min per day to practice this responsive communication and to keep a diary of when, where and for how long you manage to do this • The Health Visitor explains to you how shared book reading benefits your child • You have a wristband—like a fit bit—which records how much you say to your child and you get a daily report • You and the Health Visitor make a plan together about the best times in the day and activities to practice this responsive communication • You and the Health Visitor reflect on how things have gone this week and what you might change • You are given fridge magnets to help you to remember how to be a responsive communicator with your child • You are asked to teach a close family member how to be a responsive communicator and to support you • Over the weeks you create a library of you and your child playing and communicating to look back over and share with your family • You are helped to identify resources in your local community where you can get help and advice, meet other parents and where your child can experience play with other children
Focussed stimulation	1) Work with a practitioner to choose a language goal for your child—this can be target sentences or target words. 2) Identify activities in the day to use that target sentence or target words with your child 3) Set up play activities to encourage your child to use the target words or sentences. 4) Ask your child to follow instructions and copy you saying these words or sentences.	• You watch the professional show you how to play with your child using focussed stimulation • While you play with your child using the recommended ‘focussed stimulation’ you are videoed and then you and the professional look at the video together and see what you might change • You attend a group at a community centre or library to work with other parents to learn the recommended techniques for focussed stimulation—you problem solve how to create play situations to encourage certain kinds of words and sentences • You are helped to plan games to play with your child to encourage certain kinds of words and sentences • You agree goals to work on over the next two weeks—choosing games to play and how often to try them • You are asked to think back and reflect on how well you have done over the past fortnight and think about things you might change • You receive toys in the post with a newsletter explaining how to play to encourage certain target language structures appropriate for your child’s age

**Table 4:** Descriptions of family ‘persona’ used to elicit practitioners implicit decision-making processes

Persona	
Sam and Joe have 4 children. The child you are visiting has 2 older brothers and a younger sister aged 4 months. The family lives in social housing and receives benefits. Sam works full time in a bar near home. Joe works 2 or 3 h a week for a cleaning company. Joe and Sam both left school at sixteen. Most of their extended family live nearby. They try to manage their shifts so as not to have any additional childcare and call on family and friends.	
Lee has two children under the age of 4; you are visiting the younger child. Lee left school at 16 years, is currently not working and is in receipt of benefits. The family is living in a one-bedroom flat far away from Lee’s extended family. You are concerned about Lee’s mental health. When you observe the family, you feel Lee’s response to her children is very inconsistent, sometimes responsive and other times less so.	
Alex and Lesley live in an estate of privately owned houses. Alex works full time as a head teacher at the local school, and Lesley is currently not working. They have two children, and you are visiting their youngest child who had low birth weight and spent a short time on SCBU. They are worried about their child’s behaviour. Her attention seems to flit from one toy to another, and she can be hard to manage if she does not get her own way. Lesley seems very distressed when describing day-to-day life.	
Susie is a first-time parent who was pregnant when she left school. She is not in paid work and receives benefits. She lives with her parents in social housing. Her parents are also both long-term unemployed. She has a large community of friends and family nearby.	
Ivory and Chidi have 5 children aged between 1 and 13 years, and you are visiting their second youngest child with an interpreter. Both Ivory and Chidi speak very little English and are currently not working. The estate where they live has poor transport links and few community services. You are concerned about Ivory’s mental health.	
Nehal and Gurpreet both work full time: Nehal as a police officer and Gurpreet as an IT technician. You are visiting their first child. They have no extended family nearby and rely on a mix of a childminder and private nursery for childcare.	

### Stage 3 methods

Seven co-design workshops with 22 participants were facilitated by two members of the study team at each workshop (CM and either RW or SR). Practitioner workshops began with questions to understand the local pathway for children with speech, language and communication (SLC) needs. Parent/caregiver workshops began with an exploration of the participants’ motivation for attendance which also uncovered their experiences of the local pathway. A co-design activity was then completed which involved participants being presented in turn with the candidate target behaviours (e.g. shared book reading) and intervention techniques (e.g. diary completion) identified in Stage 2 (Table 3). Barriers and enablers to and acceptability of adopting the target behaviour or implementing the intervention technique were then explored. Paper-based workshop materials were used to stimulate discussions and helped to scaffold and steer the topics covered. These materials were manipulated and annotated during discussions by the study team and participants. All workshops were audio recorded. The above yielded the raw data for analysis: verbatim transcriptions of the workshops, annotated workshop materials, field notes and team reflections.

Methods for intervention development, described by Michie and colleagues and often referred to as the Behaviour Change Wheel and Capability, Opportunity, Motivation, Behaviour (COM-B) models, were followed [41, 71]. These methods ensure a systematic, theory-driven approach to intervention design providing methods and frameworks for (1) a thorough assessment of the behaviour to be targeted (the Behaviour in the COM-B model); (2) precise and detailed analysis of what is needed for individuals to change that behaviour (Capability, Opportunity and Motivation factors); (3) identification of the types of intervention functions and techniques which have proven efficacy in bringing those *specific* types of change about (Theoretical Domains Framework and Intervention Functions); and (4) determination of the best methods to implement those intervention functions and techniques, whether that be through service provision, communication and marketing, fiscal measures, regulation, and so on (the Policy categories section of the Behaviour Change Wheel). A four-step deductive analysis was completed to (1) map identified barriers and enablers and intervention techniques identified in the workshops and the research papers to the Theoretical Domains Framework (TDF) (e.g. Physical skills, Knowledge, Memory, Attention and Decision processes); (2) map the identified theoretical domains to candidate intervention functions (e.g. Training, Education, Environmental Restructuring); (3) map the intervention function to candidate ‘policy categories’, that are the candidate platforms through which a specific intervention function can be delivered effectively (for example, ‘Fiscal Measures’ may be appropriate for environmental restructuring function to reduce alcohol intake, ‘Communications and Marketing’ may be appropriate for Education to reduce smoking, and ‘Service Provision’ may be needed for training to increase physical activity); and (4) intervention functions identified as relevant to the TDF and barriers/enablers but judged not to be appropriate to the intervention context were discarded (e.g. Restriction, Coercion).

Field notes and team reflections identified that several qualitative, socio-relational aspects of intervention delivery were being identified by participants as crucial to intervention success. Inductive analysis was therefore also completed to analyse the verbatim transcripts to identify themes which were not determined a priori, which emerged as important to intervention design.

Finally, a paper model of a proposed intervention was developed based on the identified intervention functions and policy categories. Judgement was used to determine which were the most relevant to the intervention. This judgement was informed by comments on acceptability from the co-design workshops at Stage 3, discussion with the wider team, knowledge gained of the contextual factors of importance through PPI, and themes of acceptability from the parallel study regarding parent/caregiver perspectives on the ELIM/developmental review.

### Stage 3 results

Table 5 presents the results of the deductive analysis mapping the barriers, enablers and techniques to the theoretical domains framework; the theoretical domains framework to the intervention functions; and the intervention functions to the ‘policy categories’. Table 6 presents the key finding from the deductive and inductive analyses and previous methodological stages which informed the development of a prototype intervention for further development and evaluation in Stage 5. These were

- Identified target behaviours for the intervention;
- Appropriate intervention contexts;
- Barriers and enablers to the targeted behaviour change which may exist across families;
- Candidate intervention functions;
- Candidate intervention delivery level/ policy categories;
- Factors for equitable, acceptable and practicable intervention delivery.

**Table 5:** Mapping data to the Theoretical Domains Framework, intervention functions and policy categories

COM-B	TDF	Specifics of barriers and enablers of relevance to the domain	Intervention function	Policy categories
Physical capability	Physical skills	• Skill development—learn the skills of how to share a book; follow a child’s lead, etc. • Skill development—adult literacy to be able to share a book • Skill development—how to share SPECIFIC books, what questions to ask, etc.	Training	Service delivery
Psychological capability	Knowledge	• Procedural—know how to share a book; follow a child’s lead, etc., play and read • Knowledge—know which behaviours are important and why • Knowledge—what is an age-appropriate book • Knowledge—what kinds of questions could they ask in shared book reading?/How would they support child’s enjoyment in reading?	Education	Communication/ marketing service provision
	Cognitive and interpersonal skills			
	Memory, attention and decision processes	• Learn how to reason about what to change to do target behavior (e.g. through watching other people on video)	Training Environmental restructuring Enablement	Service provision Service provision
	Behavioural regulation	• Self-monitoring; action planning	Education Training Modelling Enablement	Communication/marketing Service provision
Physical opportunity	Environmental context and resources	• Material resources provided—books & toys • Cues and reminders to carry out the behaviours (e.g. fridge magnets, phone calls). Bookstart was a reminder as well as a resource • Material resources opportunities for play through attending playgroups/drop-ins. • Time available for busy family, mobilising wider family, piggyback on routines • Availability of age-appropriate books in Home Languages • Access to transport to access wider support • Accessing their rights for paid childcare—need support? • Special, concrete materials can help mobilise wider family resource • Need to work for full-time working parents as well as non-working	Training Restriction Environmental Restructuring Enablement	Service provision Fiscal measures Environment/social planning
Social opportunity	Social influences	• Modelling—seeing others doing it • Social norms; group conformity, group norms, social support, group identity, modelling (e.g. in parent groups) seeing others do it • Social support—getting wider family involved • Group norms—may be cultural differences in adult–child interaction patterns • Social support—importance of faith communities • Social support—cannot be stigmatising and needs to be intrinsically motivating/fun	Restriction Environmental restructuring Modelling Enablement	Communication/marketing Service provision Fiscal measures Environment/social planning
Reflective motivation	Professional/social role and identity	• Social role—parents want to do the best for their child	Education Persuasion Modelling	Communication/marketing Service provision
	Beliefs about capabilities	• Self-esteem, belief about own capabilities, perceived competence (e.g. training others) • Some parents may believe they are “doing it all” (need to be challenged?)	Education Persuasion Modelling Enablement	Communication/marketing Service provision
	Optimism	• Optimism—self efficacy through video of progress	Education Persuasion Modelling Enablement	Communication/marketing Service provision
	Beliefs about consequences	• Believe advantaging children if give them technology whilst underestimating value of own interactions • Want to do the best for their child—but need simple messages explaining benefits of specific behaviours to their child • Parent believes child cannot do it—sees HV do it and changes their views • Parent may believe that what they do will not make a difference. • Parent may believe watching a story on the television is the same as sharing a book	Education Persuasion Modelling	Communication/marketing Service provision
	Intentions	• Develop intentions to do the behavior—agree to try • Maintain stable intentions	Education Persuasion Incentivisation Coercion Modelling	Communication/marketing Service provision
	Goals	• Set goals—describe and identify concrete time and contexts to do behaviours • Action planning • Choose goal and time of day and make a very specific time and context to try something new • Know that you will be reviewed—have check in a good motivator	Education Persuasion Incentivisation Coercion Modelling Enablement	Communication/marketing Service provision
Automatic motivation	Reinforcement	• e.g. Through video of child’s progress • Having a go and seeing it work is best reinforcement • Video could reinforce feelings of hopelessness—inability • Pleasure gained from sharing ‘special toys’ • Books/gifts much more rewarding than info giving leaflet—more likely to engage	Training Incentivisation Coercion Environmental restructuring	Communication/marketing Service provision
	Emotion	• Embarrassment/discomfort to try new behavior • Overwhelm • Fear of exposure as not having skills themselves	Persuasion Incentivisation Coercion Modelling Enablement	Communication/marketing Service provision

Analysis is based on guidance and resources in Michie, S., L. Atkins, and R. West’s, The behaviour change wheel: a guide to designing interventions. 2014, Surrey: England: Silverback Publishing. Strike through (i.e. Incentivisation) indicates an intervention function identified as relevant to the TDF and barrier/enabler but judged not to be appropriate to the intervention context

**Table 6:** Summary of key results feeding forward to final intervention design

**Target behaviours for the intervention**	Use a chosen responsive interaction behaviour for 10–15 min every day from the following list: • Get down to your child’s level • Follow your child’s lead and interests • Pause and wait for your child to show you what they are interested in • Listen, watch and respond to their communication—this can be words, points, sounds or movements • Describe what your child is doing or looking at—imagine what they are thinking and feeling and say that • Show them you are having fun, and use an interesting voice • If they do communicate, copy what they say or mean to say, and add a word • Try to use fewer questions, and instead describe what is happening. • When you do ask questions try to keep them open—where, who, when and why rather than Yes/No questions
**Appropriate intervention contexts**	In daily routines chosen by parents/caregivers • Bath time • Getting out and about in the pram to the shops or park • Breakfast, lunch or tea time • Nappy change time • Playing with toys • Sharing books • At the library or toddler group • Bedtimes • Any other ‘together time’
**Barriers and enablers to the targeted behaviour change**	Listed in Table 7
**Intervention functions**	Primary functions—Training, Enablement, Modelling, Persuasion Additional function—Environmental restructuring^a^
**Intervention delivery level/ policy categories**	Primary category—Service delivery Secondary category—Communications/marketing Additional category—Fiscal and environmental/social planning^a^
**Factors for equitable, acceptable, practicable intervention delivery**	Tailoring Language and principles of shared decision-making Modelling Alliance and trust between practitioners and parent/caregivers Inclusive Motivating resources and approach Aligned to current services

^a^ Of specific relevance to families where there is a need to tackle barriers with respect to physical and social opportunities

A paper prototype intervention was developed instantiating these features into a proposed model for workshop purposes.

### Stage 4 methods

Six co-design workshops were conducted with 33 participants, facilitated by two members of the study team. Workshops at this stage involved a ‘walk through’ of the phases of the proposed intervention with paper ‘mock-ups’ and descriptors of materials and processes. Workshop resources included triggers to comment on the acceptability and feasibility of the proposed model eliciting suggestions as to how the phases should be presented and what materials should be used. The intervention model was refined and improved iteratively between workshops with modified materials presented at each site in light of previous workshop findings. Discussions were audio recorded, and participants manipulated and annotated paper materials during discussions. Verbatim transcripts of discussions were subjected to a content analysis to check and challenge the final model produced. The model was also ‘walked through’ with a subgroup of the study team part way through the participant data collection (CM, JL, VG, SR). Verbatim transcriptions were also analysed inductively to supplement the previous analysis regarding the qualitative aspects of intervention delivery which would be crucial to intervention success.

### Stage 4 results

The following presents key learning from the phased methodology and a final intervention model derived through the synthesis of the evidence, and views of stakeholder and expert practitioners. We first report the finding of the inductive data analysis relating to two key themes: (1) Parent and practitioner views on the need for an intervention and (2) Key characteristics required for acceptable, equitable and practicable intervention delivery. We then present the final conclusions from the deductive analysis identifying (3) the acceptable target behaviours, contexts and intervention techniques; (4) the barriers and enablers to the identified behaviours across families and (5) the identified acceptable intervention functions and policy categories. Finally, we synthesise the above findings into (6) the final intervention model for practice and implementation.

#### Parent and practitioner views on the need for an intervention

Using NPT, the data from practitioners suggested that the PHE SLC ‘train the trainer’ programme was supporting practitioners to do the work of coherence/sense-making and participation/engagement which is required to embed speech, language and communication interventions into practices at the 2–2½-year-old review. That is, practitioners had an appetite and indeed an enthusiasm to complete this work, see it is aligning with their role and skills and had ‘bought in’ to delivering interventions to support child language development. However, we found that the next step of *enacting the intervention* was difficult for practitioners. They were not sure *precisely how* to deliver support to families and discussions of the potential provision of concrete resources was welcomed.

*Prac-WS7: “except we don’t have anything specific do we to show, that’s the thing. There’s nothing that I’m going to go back and I’m going to go in and I’m going to show this because that is what we do. There’s nothing set in stone that that’s what we use, is there? I think that’s probably a big problem because people are going back in, there’s not a definite this is a route we need to follow, is there really?”*

Parents also articulated a desire to ‘get started’ to take action that would help their child. They expressed feelings of helplessness, frustration and anger if they felt that nothing was happening, and their concerns were going unheard.

*P-C-WS5: “because…you feel like something is happening which psychologically is good rather than, “We’ll wait a year and she’ll probably start speaking….You can be proactive and do things.”*

*P-C-WS2*: *“When they did his two year one, they didn’t say, “Come back in four weeks or two weeks,” it was eight months so in that eight months we could have got something started rather than making us just leave it this late”.*

They emphasised however that provision of this intervention must not introduce delays in referring children with severe difficulties and/or broader developmental concerns to SLTs and/or paediatricians/psychologists/audiologists. Rather, it should allow those families to begin supporting their child immediately whilst waiting for specialist assessment if that were the appropriate next step.

*P-C-W5:” As long as it’s made perfectly clear to them that they just can’t be left flailing around for two or three years like they have been……And listen to parents because they know if something is wrong.”*

#### Key intervention characteristics necessary for success

##### Practitioner language and communication

It is difficult to overstate the importance of the specific language used by practitioners to talk about children’s difficulties, and what parents/caregivers could do to help support their child. Language must be avoided which implies blame and judgement. If not carefully presented, advice can elicit strong negative feelings.

*P-C WS3 : “might have thrown something at her to be honest”*

*P-C WS3: “you’ve done everything and you’ve read every book, every audio book and every study you can find online and someone says, “Have you tried talking to your child?” you just go, “I’m either going to breathe or lose it so I’m just going to go.”*

Communication should invite the parent/caregiver in as an equal in a process of shared decision-making, setting goals appropriate to *the specific family*. Experienced and skilled practitioners invite parent/caregivers to express preferences, try new behaviours and feedback and problem solve together.

*Prac-WS7: “it’s very much like they feel that you’re going in there to tell them they’re doing it wrong. It’s not about that. It’s about them learning the best way for them to do it themselves, isn’t it really*.”

*P-C-WS3: I think if she’d said, “I’m sure you’re doing a brilliant job but here’s a couple of things you might not have thought about. You could just have a look at this list, it might give you a couple of pointers,” rather than, “Right, well this is what you’ve got to be doing to make your child speak. Do you speak to your child?”*

*P-C-WS3: “I think a dialogue rather than just being told. A dialogue is good”*

##### Alliance and trust between parent/caregiver and practitioner

Relationships of trust between practitioner and parent/caregiver were vital and, if not built at this stage, then continued engagement with the intervention and therefore its success are extremely unlikely. Facilitators of alliance and trust included demonstrating interest, engagement and expertise in interaction with the child at the review; a communication style which invites partnership, dialogue, and shared decision-making; and continuity of support from the same practitioner over an extended period.

*P-C-WS5: “because the number of times I’ve told my daughter’s story”*

Continuity was also seen as being important in supporting practitioners to make correct judgements as to the barriers and enablers which might exist for a family’s ability to engage in responsive interaction and so to choose the level of support required.

##### Modelling

The role of modelling responsive interaction by the practitioner with the child was identified by both practitioners and parents. This modelling seems to fulfil several functions:

- Demonstrating the behaviour in a non-judgemental non-threatening manner
  *P-C-WS3: Well I found it useful being shown, not being dictated to but being shown and not in, “I’m now going to show you how to talk to your child,” but more just doing it naturally. You think, “Oh.” I found that really useful… I think when you’re being told this is what you’ve got to do but when you see it and you see the way the child engages with it, you see how it works, whereas when you’re just being told, “Do this, do this,” I don’t know, you’re butting your head against it a bit and you’re feeling a bit just shouted at.*
- Demonstrating the value of specific responsive interaction behaviours and the potential for the child to engage and benefit from those behaviours
  *Prac-WS7: “We model a lot of those kind of behaviours in the visit with the parents themselves but also with the children and then they see the child responding. Then they’re building their confidence up to do that themselves as well.”*
  *P-C-WS3: But I think what was an amazing light bulb moment for me is when I saw the speech and language person speaking to Gemma*^1^*, engaging and doing things and she was engaging back. It was amazing,”*
- Promoting the parent/caregiver’s trust in the practitioner

Modelling promoted trust as it demonstrated the practitioner’s skills in engaging with the child; ensured any advice given was informed by the individual child’s temperament, developmental level and needs; and facilitated joint and individualised problem solving.

##### Attractive and motivating resources

The number of information sources and media which compete for parents’ attention was mentioned several times. Practitioners identified the need therefore to design any messaging and intervention resources in a way which would capture the attention of parents and motivate them to engage.

##### Inclusiveness and accessibility

Practitioners commented on how effective they found visual resources in other aspects of their practice. These included the use of video, attractive visual resources, ‘cue cards’ and visual reminders. For inclusive and accessible practice, they emphasised that any physical resources must be ‘relatable’ and represent the range of families served by HV teams in England, require minimal literacy levels, be readily adapted to languages other than English and be designed to take account of the range of digital inequalities.

There was substantial variation across sites as to the accessibility of sources of *social support* for families, such as parent and toddler groups, and opportunities for early childhood education and care (ECEC). Barriers to access included transport in more rural communities, recent reduction in local authority provision and confidence to attend, particularly for more socially disadvantaged families, families who had concerns about their child’s behaviour and those from minority ethnic groups. The financial support for paid childcare hours is also often difficult for families to navigate with some not being sure of how to access this.

##### Fit with current services

For the intervention to be practicable and acceptable, it would need to fit into current service provision in terms of HV team models of care, early childhood education and care provision and also local onward referral pathways.

##### Tailoring

If the intervention is not tailored to the individual family and child, there is a substantial risk of it not being manageable for the family and of making them feel judged, patronised and/or set up to fail. The following explains how key components of the intervention (behaviours, contexts and intervention techniques) need to be tailored to the individual family’s context and preferences for them to engage with the intervention.

#### Acceptable target behaviours, contexts and intervention techniques

##### Behaviours

Parents/caregivers and practitioners preferred an approach which would allow them to integrate any new behaviours into their everyday routine, rather than as an additional activity. Practitioners felt that the contingent responsive interaction behaviours (Table 4) aligned well with their current practice, underlying philosophy and the messages which they provide at other reviews.

*Prac-WS4: “It has to come with their own life and the way they are and how is that going to integrate into to their lifestyle so they can make the changes”*

*Prac-WS1: “it because part of your flow of conversation rather than being told what to do. We talk about responsive feeding, we talk about responsive parenting. That word responsive comes in”*

There were substantial differences across parents in which responsive behaviours they felt they needed/wanted to try to do more frequently. It was also important that any goal was perceived to be focussed and manageable.

*Prac-WS4: I think it feels big….it needs to be broken down*

*Prac-WS4: “But it’s about choosing one or two things and not too many things…I think giving them too much and bombarding them with too many things…”*

*Prac-WS1: “it’s something they already do, and you’re not asking them to do too much. They’re not overwhelmed.”*

##### Contexts

Importantly, jumping too quickly to a specific context within which to practise these behaviours risked alienating families. For example, when considering shared book-reading interventions, families reported multiple ways in which this context could cause problems. This included parent/caregivers’ perception that it suggested that they might not know book reading was a good idea, which felt patronising, or that they did not do enough book reading, which felt judgemental.

*P-CWS1: “I’d be quite offended because I read a lot with my kids. We had this and they said, “Mum, you need to read with them.” I read with them quite a lot. I do at least four books on a night …. Then they’re saying, “Read with them. That’s why he doesn’t, you just have to read…. Yes, like it’s our fault”*

Furthermore, if book reading felt too difficult for the parent/carer either because the child was not ready or they themselves had some literacy difficulties, this would likely feel too difficult and that it was setting them up to fail.

*P-C-WS1: “Everything needs to be the way Danny likes. If I want to read a book to Danny, no, because he wants another book. If you’re reading a book to Danny, he’s like, “That’s enough.” He has enough with the book so it’s just like…I don’t want to be shouting all the time, “Danny Sit down, Danny.” I’m like, “You know what? I’m just going to let Danny when he wants it,” because I don’t want to frustrate him”*

*P-C-WS1: “So to be honest, I’m not very good at reading books but my husband has a little bit more patience with the language because it’s not my language so for me to read, I need to take… a lot of times.”*

Other parents would very much welcome support with how to share books with their child:

*P-C-WS2: “I’m not so creative so maybe if we got a sheet with questions on it, that would help a bit more.”*

It was clear that different families needed and preferred different contexts to practice the chosen intervention behaviours.

*P-C-WS5: “ I just built it into my day all the time really at the moment, when we had a moment….I just worked it in wherever we were.”*

*“On the flip side, for me, having multiple children I wouldn’t be able to work it into my daily because it’s just mental sometimes….but for me, this would be brilliant because I would go, “Actually yes, I do need to find a time in my day to focus and that will be my time. That will be when the others are in the bath, dad is bathing them. He can bath Ella and Jack and I will sit on the sofa with Archie.”*

*Prac-WS4: “I think it’s the time when they are together that is the critical time. It’s making the most of that together time”*

##### Intervention techniques

In terms of intervention techniques extracted from previous research and discussed in the workshops (Table 4), most were felt to be acceptable if their implementation could be adjusted to the family’s context, if explained appropriately, and if delivered in the context of a relationship of trust between the parent/caregiver and the practitioner. The exceptions (techniques which were considered *not* acceptable) included the parent/caregiver being videoed by the practitioner; the use of a ‘language fit bit’ which records how much the parent says to the child and gives a daily report; and teaching another family member how to be a responsive communicator. The former two bringing with them a power dynamic which was not welcomed by many families and a sense of being ‘surveilled’ and the latter raising significant difficulties with respect to family dynamics and difference of opinion as to how best to parent between partners and across generations.

#### Barriers and enablers

The work above identified the target behaviour for the intervention: *parents/caregivers increasing the frequency of use of one or more of set of responsive interaction behaviors.* The barriers and enablers to the use of responsive interaction language-promoting behaviours in the home identified in Stage 3 are synthesised and summarised in Table 7.

**Table 7:** Enablers identified as needing to be in place to engage in the target behaviour change (increase frequency of responsive interaction behaviour) organised with respect to the COM-B components and the TDF domains)

COM-B component	TDF domain and description of enablers
Capability	**Physical skills** Have skills to follow a child’s lead in play or share a book and use responsive interaction behaviours Have literacy skills to share a book
	**Knowledge** Able to choose age-appropriate books, toys and activities Know what kinds of questions to ask during book sharing/shared activities and how to follow child’s interests and respond contingently
	**Decision-making** Able to decide on what they need to change to achieve their goal
	**Regulation** Able to monitor their own use of the new behaviour and make and stick to an action plan to do it
Motivation	**Belief about capabilities and optimism** Feel they can make the change and increase the use of this behaviour Feel making the change is worthwhile and that there is scope to increase their responsiveness
	**Beliefs about consequences** Feel child will engage and so will respond or benefit Feel the chosen behaviours are best for the child and other behaviours (e.g. TV viewing) are not equally good—have reason to change Feel that what they do will make a difference
	**Intentions and goals** Have definite intention to try to increase their use of the behaviour Able to set a clear goal and create action plan for implementing it **Emotion** Do not feel embarrassed at trying new behaviour and/or have fear of exposure/being judged Do not feel overwhelmed by additional demands
Opportunity	**Physical opportunity A** Have the books and toys needed to use this new behaviour including books in home language Have access to playgroups, drop-ins or other contexts to support the use of these behaviours
	**Physical opportunity B** Have a family and/or social network to draw on to support them Have access to/making use of childcare for siblings or child
	**Social opportunity** See others in their social group using the responsive communication behaviours in a range of contexts Have a family and/or social network to also use the behaviours with their child Have opportunities for supported ‘together time’ which is intrinsically rewarding for child and parents

#### Intervention functions and ‘policy categories’

We further examined the barriers and enablers identified above together with the intervention techniques drawn from the literature which participants judged to be acceptable and feasible to identify the most relevant intervention functions and policy categories to be used in the intervention (see Table 5). These were mapped to relevant intervention functions, and Training, Enablement, Modelling, and Persuasion were identified as the most relevant functions. The main policy categories/platforms for delivery identified as relevant to our shortlist of intervention functions and theoretical domains were Service delivery and Communications and Marketing suggesting a combination of implementation approaches across health/educational services and marketing would be beneficial (Table 5).

#### The proposed intervention

**Fig. 3:**
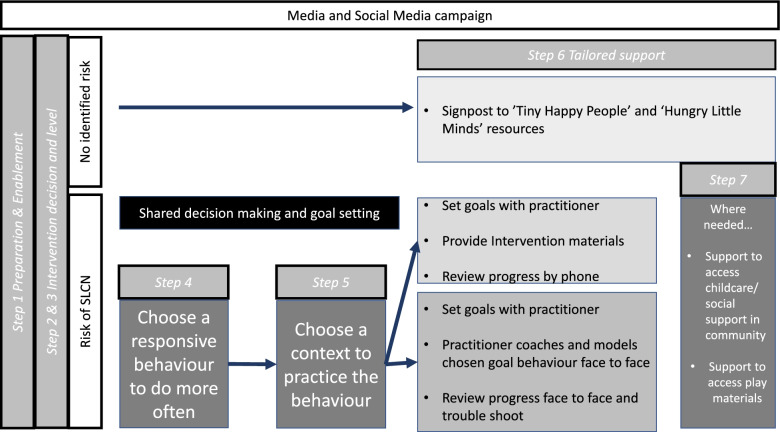
The proposed intervention model

The proposed intervention aims to empower families to act to support their child as soon as the risk of SLCN is identified, applying current best evidence in a timely manner. It aims to ensure equity of access for all children and families through tailored guidance and support (Fig. 3). The intervention does not replace local SLCN pathways but rather is designed to become coordinated with and integrated into them. It is essential that children continue to be referred for support by SLTs and other professionals where they meet local criteria for referral and receive enhanced support in their early years settings as appropriate.

*The steps in intervention delivery are as follow*:

- Step 1: Preparation
- Step 2: Decide on the need for intervention and/or onward referral
- Step 3: Choose intervention level
- Step 4: Choose a responsive behaviour to do more often
- Step 5: Choose the context in which to practice the behaviour for 10–15 min daily
- Step 6: Deliver tailored support
- Step 7: Offer optional additional support

Step 1 focusses on the preparation, which is necessary for successful shared decision-making and engagement [53, 72]. In order to address power imbalances in the practitioner–parent/caregiver relationship [18] and ‘activate’ the parent/caregiver [73], preparatory materials are needed which welcome and value the parent/caregiver’s knowledge about their child, establish the focus of the review [72] and encourage the parent/caregiver to arrive with questions and reflections. Step 2 is essential in mobilising and motivating action by the parent/caregiver and creating practitioner–parent/caregiver alliance [36]. Steps 3–5 focus on shared decision-making and goal setting. Steps 6 and 7 relate to intervention delivery. The proposed procedures, content and materials of each intervention stage including recommendations regarding the language to use and methods of presentation are described in detail in *Supplementary Materials**2**.*

##### The intervention model and its components

The goal of the intervention is to increase parents/caregivers’ use of specific responsive interaction behaviours for 10–15 min per day in a specific context, which suits the family’s resources and constraints and is part of their usual daily routine.

The intervention delivery platforms draw on the identified relevant policy categories of Service Delivery and Communications and Marketing. All families receive one of three levels of support through HV services (Service Delivery) and links to a universal media and social media campaign (Communications and Marketing) (i.e. resources already published or under development by the Best Start in Life program: ‘Hungry Little Minds’ [74] and ‘Tiny Happy People’ [75]). Two optional additional support packages may also be offered (see Fig. 3). Which level families receive and whether the optional additional support is offered is determined by the outcome from the ELIM-I measure (developed as part of this study and reported elsewhere) and also practitioner judgement as to the assets and challenges for the family and the barriers and enablers to accessing the intervention—a judgement which is guided by resources and training based on the COM-B model and Theoretical Domains Frameworks [41, 42] (see below).

*Level 1: children with no identified risk*

We recommend the framing of this review as a time to talk about setting the foundations for the child’s learning and ensuring all children reach their full potential [24, 76]. As such, all families should be signposted to available resources which provide guidance as to how to support children’s language development. This universal provision of accessible information potentially brings three key benefits. First, we know that trajectories of language development can be unstable and unpredictable between 2 and 4 years of age, and some children who appear to be developing well at 2 years may develop language difficulties later [32]. By ensuring all families are provided with appropriate resources to support them to provide an enriching language environment, we provide a ‘safety net’ for those who may not be identified at this review. Second, parents/caregivers’ perception of the value of the 2–2½-year review and their subsequent engagement with services is partly influenced by whether they learn something new at that appointment [32]; guidance on child language development could meet these preferences. Third, taking a universal rather than targeted approach brings an additional advantage of reducing the potential for stigmatisation which can be inherent in some targeted interventions. Targeted selective approaches offer intervention to groups who are more likely than others to develop a particular condition. In the case of language interventions, this is usually families living with social disadvantage. Such approaches carry the risk of unintentional stigmatisation and consequential disengagement of targeted groups [77]. This can be avoided where families see that the support is universally offered albeit with varying intensity according to need.

*Level 2: children with identified risk—self-directed approach*

This level of support is for children identified as being at risk of SLCN using the ELIM and where practitioners judge there are few barriers to the targeted behaviour change. Where barriers do exist, the practitioner judges they mainly relate to the *Capabilities* category of the *C*OM-B model (Table 7). If the child meets the criteria for SLT referral for the local pathway then this should be actioned. Practitioners discuss the need to support their child’s language development and the nature of responsive interaction. Language is carefully chosen which promotes the building of trust and engagement and avoids implications of blame or judgement (see Supplementary Materials 2). Using a shared decision-making tool, practitioners support families to choose a responsive interaction behaviour which they would like to try to do more often and identify the context and times in the day when they will be able to try this—their ‘Together Time’.

Detailed guidance is provided for the practitioner about each step (see Supplementary materials). This includes suggestions for how to support parents to sustain and adjust their interactions including review and reflection techniques, recording and aide memoire strategies (see Supplementary materials 2 for more detail).

*Level 3: children with identified risk—coaching approach with additional practitioner support*

This pathway is for children identified as potentially being at risk of SLCN using the ELIM and where practitioners judge there are a number of barriers to the targeted behaviour change, particularly in the *Motivation* and/or *Opportunity* categories of the COM-B model (see Table 7). This level uses similar approaches as level 2 above but with additional face-to-face support from the practitioner to tackle motivation and opportunity barriers to change and offer more support for knowledge and skills development. This support takes the form of coaching through modelling, practice and supported reflection and goal setting, with the practitioner offering regular visits until the parent/caregiver is confident they can integrate the behaviour into their daily routines (see Supplementary materials 2 for more detail).

*Optional additional support package 1—access to early years settings/social support*

An optional additional support package should be offered to families with barriers to behaviour change identified with respect to social opportunities and physical resources necessary for those social opportunities (see Table 6). Design and delivery of a support package to facilitate access to social opportunities will require knowledge regarding local provision and the community assets and resources, that can be mobilised. Action by the practitioner alone is not sufficient if local provision is not accessible to all families.

*Optional additional support package 2—access to age-appropriate books and play materials*

The responsive interaction behaviours targeted in this intervention do not require the provision of any specific play materials or toys. Indeed, the goal of the intervention is to support families to integrate responsive interaction into their usual daily routines. In general, no additional toys or children’s books are likely to be required. However, in some cases, where the family identifies ‘playing with toys’ or ‘sharing books’ as their preferred ‘together time’ and where the family resources are extremely limited, practitioners should consider a support package to address access to toys and books. This may involve support to access toy libraries and the local library. As in the case of ECEC provision, many barriers to access to these resources exist.

We recommend local co-design of both support packages to identify barriers and enable access to parents and toddler groups, playgroups, local libraries and toy libraries for families who need this support: those with social and physical opportunity barriers (see Table 6). Co-design work should involve all agencies involved with early years provision, those practitioners who signpost families to them and parents/caregivers and may include the development of resources to support families to use everyday materials available at home to develop play and language.

*Media and social media campaign*

Our analyses identified that in addition to the service delivery approaches we have described above, that the policy category ‘Communications and Marketing’ was also a relevant platform for delivery of support. Existing social media resources from the ‘Hungry Little Minds’ and ‘Tiny Happy People’ [33, 75] campaigns align closely to this intervention model. There was, however, a sense of being overwhelmed from some practitioners we spoke to in terms of the range and sheer volume of materials whilst others were not aware of the ‘Tiny Happy People’ campaign. There was an identified need from practitioners for help to navigate the resources and identify which might be best for which purposes. Both parents and practitioners suggested many families will not seek this information out and, in some cases, may be uncomfortable with a perceived ‘educational’ tone. The use of a range of social media platforms and active campaigns were suggested as being necessary if these messages are to reach all families of young children. We therefore recommend ‘joining up’ of this intervention with existing resources and social media campaigns so that the materials developed in this intervention clearly signpost to the high-quality resources being developed.

*Skill-mix, delivery and normalisation*

Steps 1–5 of this model (encompassing preparation, identification, tailoring and shared goal-setting) require a holistic approach to both child and parent health and wellbeing and knowledge of the family and so we recommend that the HV take the lead at these stages. The provision of tailored support and additional support packages (Steps 6 and 7) could involve a more mixed model with skill mix in HV teams or Early Years Practitioners in early years settings delivering the tailored support and/or the optional additional support packages in consultation with the HV team. For those families where coaching and additional support packages are required (Level 3) Speech and Language Therapy services could also be involved either directly or as advisors to the practitioners delivering the coaching model, depending on the configuration of the local SLCN pathway. This should be negotiated and discussed as part of the local co-design work we recommend above which will be required to develop implementation and sustainability plans for integration into local service delivery context. We recommend that for implementation and maintenance of this programme of work that an integrated team of HVs, SLTs and Early Years leads is convened and maintained to steer its introduction and safeguard its sustainability [60, 61].

## Discussion

This paper presents the findings of a rigorous intervention development methodology to design a universal intervention to promote children’s language development to be delivered at the HV 2½-year review. The study applied the most recent guidance on best practice in intervention design and co-design [34, 35, 44, 45] and was informed by relevant theory with respect to early language development and disorders [22, 46], behaviour change [41], shared decision-making [53, 55], engagement [36], acceptability [41, 51] and implementation [35, 61].

The resulting intervention (ELIM-I) focusses on supporting families to increase their use of responsive interaction behaviours [22, 46] within their daily routines and in contexts tailored to individual family circumstances [53, 55]. The risks of universal interventions widening rather than narrowing inequalities was addressed through consideration of the differing barriers and enablers which may be present for families [41]. The intervention was therefore designed to offer families a proportionate and tailored response—proportionate in that the intensity of support can increase or decrease depending on the family’s needs—and tailored such that the goals and intervention approaches are modified considering the specific assets and challenges in each family. The resulting intervention therefore meets a core principle of the modernised Healthy Child Programme—Best Start in Life: ‘universal reach and a personalised response’ [33]. Families differed significantly as to where the barriers lay to changing the targeted behaviour and in their daily routines and demands on parent/caregiver time. Many published interventions focus on specific behaviours (e.g. shared book reading) [16] or on the development of knowledge and skills, and do not consider factors of motivation (such as feelings of self-efficacy and confidence to succeed) or social or physical opportunities [19, 78]. Interventions which are not tailored to the specific barriers and enablers present for each are likely not only to be ineffective, but also risk alienating families and damaging the potential for engagement with services [36]. Inappropriate advice risks families feeling blamed, judged, patronised, or set up to fail [79]: *how* an intervention is delivered can ‘make or break’ its success. This intervention therefore draws on theories of shared decision making, patient activation and engagement and partnership, to embed strategies which engender successful collaborative partnership in its design [53, 55]. Although these characteristics are often viewed as core to HV practice [80], our findings suggest families do not always experience them and, as a result, sometimes relationships break down.

Perhaps most important to this alliance is the language used by practitioners to talk about children’s difficulties, and what parents/carers could do to help support their child. Concrete, shared decision-making tools can scaffold practitioner–patient conversations to enable communication which addresses power imbalances, acknowledges families’ strengths and invites equal participation [55, 80]. Implementation will likely also require training in the use of the tools advocated here [59]. We also recommend the use of preparatory materials for ‘patient activation’ prior to attendance at the review appointment which can go some way to addressing power imbalance and hence promote dialogue [53, 73]. Particular care must be taken that discussions with parents/carers do not imply that their interaction style or the time they spend interacting with their child has caused the language difficulties they are experiencing. It can be difficult to understand and to explain that although *changing* your interaction style can improve your child’s language development that your interaction style has not *caused* their language difficulties [81, 82].

The views of both practitioners and parents suggest any intervention must maintain their sense of agency, enabling practitioners to provide a responsive service and for parents to begin to address the needs of their children. It was very clear that any such support for the family must not create a delay to access to more specialist Speech and Language Therapy Support for children with more severe difficulties and/or signs of broader developmental concerns. The degree to which SLT and HV services are ‘joined up’ and have agreed and clear co-working and referral pathways varies substantially across the UK. To deliver ELIM-I and support all children’s language development in a given locality, it is clear that collaboration between these services is vital. Further ‘joining up’ is required to tackle the identified barriers of physical and social opportunity, which are best addressed through access to Early Childhood Education and Care Settings, particularly those which focus on the provision of support for the family as whole. Again, access to these varied across our sites, a picture mirrored across the UK reflecting the reduction in spending on early preventative services in 2010 and the move away from universal provision to more targeted approaches [83]. Successful delivery of the ELIM-I intervention would necessarily require commissioning and service delivery to be integrated across all the different professionals involved—health visitors and their teams, speech and language therapists and early years practitioners. This integration work also fulfils the NPT process of *enacting* wherein the innovation becomes material practice through practitioners’ operationalising the innovation into their own specific context, increasing the potential for it to become normalised practice [60].

### Strengths and limitations

This study followed recent guidance for successful intervention development. The iterative methodology served to integrate current best evidence with stakeholder preferences and rich contextual information regarding the context within which the intervention would be delivered. Extensive stakeholder engagement and co-design workshops across a diverse range of sites served to inform the final intervention design. The advent of COVID-19 restrictions in the last phase of data collection meant that our parent/caregiver participant sample was not as diverse, in terms of linguistic and cultural backgrounds as we would have hoped. In the next phase of piloting and implementation of the ELIM-I, it will be essential to ensure the views and experiences of a broad demographic of families are solicited.

This study delivered detailed guidelines for the delivery of the ELIM-I intervention. Superficially, the ELIM-I intervention is a simple one: supporting parents/caregivers of children at risk of speech language or communication needs to increase their use of responsive interaction behaviours with their child. However, the need for proportionality, tailoring and collaborative partnerships makes successful delivery to the requisite level of fidelity for intervention effectiveness a complex task. Quality improvement, especially across complex, multi-professional, multi-agency systems, is rarely easy. Innovation, such as the development of the ELIM-I is only the first step. For successful implementation of this innovation to be achieved, further development and scientific evaluation are required, and clearly, further studies to pilot it in a range of contexts and evaluate its efficacy are essential [60, 84].

### Next steps

Embedding health care and educational innovations into routine practice is not straightforward and requires explicit planning [61]. Applying NPT, our work identified that SLC training for HVs has and is supporting practitioners to do the work of coherence/sense-making and participation/engagement which is required to embed SLC interventions into practices at the 2–2½-year-old review. That is practitioners have ‘bought in’ to delivering interventions to support child language development. However, the next NPT step required for successful and sustainable implementation, that is *enacting* the intervention, remains difficult. Whilst the ELIM-I protocol provides further guidance, it is yet to be tested in practice, and, in its current form, as guidance rather than as material objects and/or local policy, there are risks with respect to its potential for successful implementation [84, 85]. Efforts must focus on the NPT stages of enacting and appraisal if the innovation is to be sustained and delivered across contexts with the required level of fidelity to the original protocol for the potential benefits to be realised [60, 61]. Practitioners are likely to require additional support and resources to enable the ELIM-I to be implemented successfully and in a manner which will narrow rather than widen inequalities through offering truly proportionate and tailored support appropriate to each family. This would include intervention materials, including preparatory letters, shared decision-making tools, video modelling resources, aide-memoires, etc., for use with a range of families and communities; training resources to ensure HV teams have the appropriate skills to model contingent responsive interaction and can make appropriate judgements regarding tailoring; and audit and reflection tools to enable ongoing appraisal and hence maintenance of the innovation in practice.

Further work is also needed for ELIM-I to be accessible to families from a range of linguistic and cultural backgrounds. We recommend further work to develop and pilot a manualised program with standardised intervention resources and guidance for local implementation and policy development. Strategies to support successful implementation with proven efficacy include the development of simple, evidence-based, accessible and visually clear and appealing resources; the use of decision support systems, checklists and digital tools, and context-specific standardised protocols [86]. Furthermore, multi-professional collaboration and the development of local consensus groups have been shown to improve implementation [87, 88].

The response of services to the COVID-19 pandemic have underlined the diversity in service provision which exists across the country for children in the first 1001 days and the benefits which can be realised when early years settings and HV services collaborate to support vulnerable families [89]. Furthermore, the DfE is ambitious to leverage the skills, knowledge, and capacity of the whole children’s workforce to give children the ‘Best start in Speech Language and Communication’. To date the ELIM-I development has focussed, in the main, on HV teams. Whilst the key principles of the ELIM-I are likely to readily translate to early years settings its implementation must be contextualised. Further work is needed to adapt the ELIM-I for use in early years settings to facilitate inter-agency collaboration.

A fundamental shift for services during the pandemic has been the move to remote support using digital tools. There is an acknowledgement amongst professionals and families of both benefits and harms from this shift. For some families, remote services are welcomed as convenient, lower cost and accessible whilst for others, the ‘digital divide’ makes them completely inaccessible. Importantly, for families with the highest level of need, remote services do not support the necessary development of professional and parent–caregiver trust and alliance for successful support to be delivered [89].

Digital remote delivery therefore will never be a panacea for service delivery to support families of children in the early years. However, within a matrix of differing tailored support, digital delivery offers the potential for convenient and economical delivery for *some* families, potentially freeing up resource for face-to-face support for those with the greatest need for specialist practitioner support. Development of methods to deliver ELIM-I digitally therefore could bring potential benefits with respect to further tailoring and personalised care, and possible cost savings. In addition, they ensure services are robust to future pandemic or other ‘shocks’ to services for children and families and have the potential to enable multi-disciplinary collaboration.

## Conclusions

It is possible to develop a universal intervention for use by HVs at the 2–2½-year review to promote children’s language development which parents and practitioners judge would be acceptable and feasible in practice. For such an intervention to be equitable and to promote engagement and partnership, it must be proportionate, varying in intensity of support, tailored, such that goals and intervention approaches address the specific barriers and enablers in each family, and must address power relationships through shared decision-making, patient activation and strength-based approaches.

## Supplementary Information

**Additional file 1.** Stage 1 detailed methods and results.

**Additional file 2.** The intervention presentation, content and materials for each step of the intervention.

**Additional file 3.** GUIDED checklist.

## Data Availability

The datasets generated and/or analysed during the current study are not publicly available due to the ethical approvals in place making data sharing non-permissible. Participants did not give permission for data sharing. All qualitative data collection materials are available from the first corresponding author on reasonable request.

## Abbreviations

HV: Health visitor
ELIM-I: Early Language Identification Measure and Intervention
SLCN: Speech, language and communication needs
SLC: Speech, language and communication
COM-B: Capability, Opportunity, Motivation, Behaviour
ASQ-3: Ages and Stages Questionnaire 3
DfE: Department for Education
PHE: Public Health England
HCP: Healthy Child Programme
APEASE: Acceptability, Practicability, Efficacy, Affordability, Safety and Equity
PPI: Public Patient Involvement
R&D: Research and development
REC: Research Ethics Committee
CNN: Community nursery nurses
SLT: Speech and language therapist
ECEC: Early childhood education and care

## Footnotes

1 All names in quotes are pseudonyms.
