# Supplementary material for: ‘Making the most of together time’: development of a Health Visitor–led intervention to support children’s early language and communication development at the 2–2½-year-old review
Source: Pilot Feasibility Stud. 2022 Feb 8;8:35. doi: 10.1186/s40814-022-00978-5 (PMC8822642; doi:10.1186/s40814-022-00978-5)
Supplement: Supplementary file 1 — Additional file 1. Stage 1 detailed methods and results. [file 40814_2022_978_MOESM1_ESM.pdf]

Supplementary materials 1: Stage 1 detailed methods and results

Stage 1: Methods

Four scoping and systematic reviews of pre-school interventions with oral language as an outcome had recently been completed by members of the study team or were in progress. To maximise efficiency studies were identified through these four reviews and two additional reviews known to the team [1-6]. Original research studies were identified and sourced from those reviews which had oral language as the outcome, involved parents as the agents of intervention and included children aged between 12 and 36 months. This yielded 27 papers. Additionally, the EIF guidebook of early intervention programs that have been evaluated and shown to improve outcomes was consulted [7] and 2 relevant papers were identified. A workshop was then conducted with the study expert team which involved 1) an appraisal of the quality and relevance of the available evidence to the service delivery context; 2) identification of additional papers to consider and 3) potential barriers and enablers for families when accessing the identified interventions.

Stage 1: Results

Final list of papers of effective interventions used to extract details of potential intervention target behaviours and intervention techniques

Intervention papers
1. Arnold, D. H., Lonigan, C. J., Whitehurst, G. J., & Epstein, J. N. (1994). Accelerating Language Development Through Picture Book Reading: Replication and Extension to a Videotape Training Format. *Journal of Educational Psychology, 86*(2), 235-243.
2. Buschmann, A., Jooss, B., Rupp, A., Feldhusen, F., Pietz, J., & Philippi, H. (2009). Parent based language intervention for 2-year-old children with specific expressive language delay: a randomised controlled trial. *Archives of disease in childhood, 94*(2), 110-116.

3. Christakis, D. A., Zimmerman, F. J., & Garrison, M. M. (2007). Effect of block play on language acquisition and attention in toddlers: A pilot randomized controlled trial. *Archives of Pediatrics and Adolescent Medicine, 161*(10), 967-971.
4. Cooper, P. J., Vally, Z., Cooper, H., Radford, T., Sharples, A., Tomlinson, M., & Murray, L. (2014). Promoting Mother-Infant Book Sharing and Infant Attention and Language Development in an Impoverished South African Population: A Pilot Study. *Early Childhood Education Journal, 42*(2), 143-152.
5. Cronan, T. A., Cruz, S. G., Arriaga, R. I., & Sarkin, A. J. (1996). The Effects of a Community-Based Literacy Program on Young Children's Language and Conceptual Development. *American Journal of Community Psychology, 24*(2), 251-272.
6. Gibbard, D., Coglan, L., & MacDonald, J. (2004). Cost-effectiveness analysis of current practice and parent intervention for children under 3 years presenting with expressive language delay. *International Journal of Language & Communication Disorders, 39*(2), 229-244.
7. Garcia, D., Bagner, D. M., Pruden, S. M., & Nichols-Lopez, K. (2015). Language Production in Children With and At Risk for Delay: Mediating Role of Parenting Skills. *Journal of Clinical Child and Adolescent Psychology, 44*(5), 814-825.
8. Huebner, C. E. (2000). Promoting Toddlers' Language Development Through Community-Based Intervention. *Journal of Applied Developmental Psychology, 21*(5), 513-535.
9. Landry, S. H., Smith, K. E., Swank, P. R., & Guttentag, C. (2008). A Responsive Parenting Intervention: The Optimal Timing Across Early Childhood for Impacting Maternal Behaviors and Child Outcomes. *Developmental Psychology, 44*(5), 1335-1353.
10. McGillion, M., Pine, J. M., Herbert, J. S., & Matthews, D. (2017). A randomised controlled trial to test the effect of promoting caregiver contingent talk on language development in infants from diverse socioeconomic status backgrounds. *Journal of Child Psychology and Psychiatry and Allied Disciplines, 58*(10), 1122-1131.

11. Lim, Y. S., & Cole, K. N. (2002). Facilitating first language development in young korean children through parent training in picture book interactions. *Bilingual Research Journal, 26*(2), 367-381.
12. Love, J. M., Kisker, E. E., Ross, C., Constantine, J., Boller, K., Chazan-Cohen, R., . . . Vogel, C. (2005). The effectiveness of early head start for 3-year-old children and their parents: Lessons for policy and programs. *Developmental Psychology, 41*(6), 885-901.
13. Mendelsohn, A. L., Dreyer, B. P., Flynn, V., Tomopoulos, S., Rovira, I., Tineo, W., . . . Nixon, A. F. (2005). Use of videotaped interactions during pediatric well-child care to promote child development: A randomized, controlled trial. *Journal of Developmental and Behavioral Pediatrics, 26*(1), 34-41.
14. Olds, D. L., Robinson, J., O'Brien, R., Luckey, D. W., Pettitt, L. M., Henderson Jr, C. R., . . . Talmi, A. (2002). Home visiting by paraprofessionals and by nurses: A randomized, controlled trial. *Pediatrics, 110*(3), 486-496.
15. Roberts, M. Y., & Kaiser, A. P. (2011). The effectiveness of parent-implemented language interventions: A meta-analysis. *American Journal of Speech-Language Pathology, 20*(3), 180-199.
16. Whitehurst, G. J., Arnold, D. S., Epstein, J. N., Angell, A. L., Smith, M., & Fischel, J. E. (1994). A Picture Book Reading Intervention in Day Care and Home for Children From Low-Income Families. *Developmental Psychology, 30*(5), 679-689.

Systematic Reviews Consulted

1. Law, J., et al., *Early Language Development: Needs, provision, and intervention for preschool children from socio-economically disadvantage backgrounds.* 2017, London: Institute of Education.
2. Law, J., et al., *Parent-child reading to improve language development and school readiness: A systematic review and meta-analysis.* 2018: Newcastle University and Queen Margaret University.
3. CRE-CL and CfCCH, *Every Toddler Talking (Phase 1) Final report*. 2015, MCRI: Melbourne.
4. Axford, N., et al., *The best start at home*, in *Early Intervention Foundation Evidence*. 2015, Early Intervention Foundation: London.
5. Asmussen, K., et al., *Foundations for life: what works to support parent child interaciton in the early years*. Evidence. 2016: Early Intervention Foundation.
6. Levickis, P., et al., *A review of interventions to promote language development in early childhood*, in *Language Development: Individual Differences in a Social Context*, J. Law, C. McKean, and S. Reilly, Editors. in press, Cambridge University Press: Cambridge.
7. Early Intervention Foundation. *The EIF guidebook*. [cited 2019; Available from: https://guidebook.eif.org.uk/.
