# Supplementary material for: ‘Making the most of together time’: development of a Health Visitor–led intervention to support children’s early language and communication development at the 2–2½-year-old review
Source: Pilot Feasibility Stud. 2022 Feb 8;8:35. doi: 10.1186/s40814-022-00978-5 (PMC8822642; doi:10.1186/s40814-022-00978-5)
Supplement: Supplementary file 2 — Additional file 2. The intervention presentation, content and materials for each step of the intervention. [file 40814_2022_978_MOESM2_ESM.pdf]

Supplementary Materials 2: The intervention presentation, content and materials for each step of the intervention

Intervention Step	Presentation and Content of the Intervention Step	Intervention Materials
**1. Preparation and enablement**	The Personal Child Health Record to contain<br>• an introduction to the concept of 'language as a foundation for learning'<br>• descriptions as to what to expect in terms of language and communication milestones between 1 and 3 years<br>• QR code/ web links to the 'Tiny Happy People' and 'Hungry Little Minds' resources.<br>The letter inviting parent/caregiver to the 2-2½ year review will include<br>• a summary version of the detail above<br>• information about what to expect at the review<br>• QR code links/web address to video explaining the processes involved and what they might like to ask<br>• trigger questions to initiate the process of reflection regarding their child's language and communication development.<br>• suggestions for ways to prepare "Here are some ideas of ways that you can	Personal Child Health Record: Language as a foundation to learning; language and communication milestones, QR codes/web address<br><br>'Tiny Happy People' resources curated as most relevant to the 2-2½ year review<br><br>Review invitation: Summary of above, QR codes/web address, things to think about before you come to the review<br><br>Video of what to expect at the 2-2½ year review and things you might want to ask.

	prepare for your conversations with the HV"	
**2. Intervention decision**	The results of the ELIM-S are used to determine whether the child is at increased risk of SLCN and, if so, whether they meet the criteria of the local SLCN support pathway for onward referral to SLT or other support services.	
**3. Choose intervention level**	*Children with no identified risk of SLCN receive Level 1 and jump to Step 6*<br><br>Children with identified risk of SLCN: the practitioner reflects on whether Level 2 or Level 3 intervention is most appropriate and whether optional additional support is needed. Practitioners will be given training in this and access to a decision support tool to use alongside knowledge of family and information gathered through ELIM to make this choice.<br><br>The practitioner discusses how specific kinds of talk and interaction 'turbo charge' children's language development. They explain how some children find it harder than others to pick up language and communication. For these children we need we need to become 'super communicators' and increase our responsive communication to help	COM-B Barriers and Enablers rubric: Practitioners will be trained to consider the Barriers and Enablers on the rubric (Table 6) and how these map to intervention level.<br><br>Intervention level mapping: Decision support tool summarising how Barriers and Enablers map onto intervention levels and options for additional support<br><br>Video: 'Super communicator' message reinforced in 2-3 minute video of

	them to learn from us<br><br>*Families offered Levels 2 and 3 continue to steps 4 and 5*	parent/caregivers modelling responsive communication in everyday contexts - with voice-over pointing out and naming the responsive behaviours
**4. Choose responsive behaviour**	The practitioner shows a short video of a parent/caregiver interacting with their toddler and engaging in responsive communication, which has, some of the behaviours tagged and explained.<br>The practitioner says that they are sure the parent/caregiver is doing lots of these behaviours already reinforcing the idea that some children need us to be 'super communicators' and 'dial up' these behaviours for them to learn from us.<br><br>The practitioner shows the parent/caregiver a set of cards, which list and illustrate responsive behaviours, talks them through to establish shared understanding and sorts them into piles of those they might or might not want to try.<br><br>The practitioner asks the parent/caregiver to choose one behaviour they would like to	Video: as above<br><br>Shared decision-making support tool: responsive communication cards with one behaviour described on each and a picture to support understanding of those behaviours

	try to do more to help their child's language and communication development.	
**5. Choose context**	Talk through how every family has different rhythms to the day and different times of the day when they might have the time or energy or help from others to be able to tune in to their child's communication. Ask the parent/caregiver to reflect on when might be their best time for 'Together Time'. Explain this can be based on what the child finds interesting, when they have help from a partner or friend, when they and their child are least tired, when the household is less busy and distracting - whenever they find they are most able to focus on their child<br><br>Show the parent/caregiver the picture list of possible situations and see if they feel any would work for them – support them to rule out those, which won't work and think of ones, which they feel they could try.<br><br>Show the parent caregiver the reverse page which includes copies of the responsive behaviour cards and a space to write a 'Together Time' goal – support the parent to fill this in with the chosen behaviour and chosen context or 'together time'	Shared decision-making and goal setting tool: 'Together time' picture list. On the reverse responsive behaviour picture list reproducing the cards from the previous step. A place to write in the agreed goal

**6. Deliver tailored support**	**Level 1. Children with no identified risk**<br>Families are signposted to available resources which provide guidance as to how to support children's language development<br><br>**Level 2. A self-directed approach**<br>Discuss with the parent/carer their preferred methods for:<br>• being reminded to try this every day – e.g. using a paper diary, a reminder on their phone, a text message from an automated texting system etc.<br>• reflecting regularly on how things are going – e.g. using a paper diary, making audio recorded notes on their phone, texting their HV team.<br>Encourage modelling, review and reflection activities<br>• leave 'exercises' encouraging families to look on Tiny Happy People website<br>• Provide motivational materials e.g web resources, which the parent/caregiver can explore, which provide motivational information about why responsive interaction is so important and modelling that it can be a fun way to connect with their child.	Diary /self-monitoring and reflection tool<br><br>Resources leaflet: details of relevant Tiny Happy People resources with suggested reflective activities to support active engagement and application to family's own context.<br><br>Concrete reminders – based on the responsive behaviour cards – of the parent/caregivers goal for the week to put on their fridge/noticeboard.

	• If this feels like too much information for the family, consider sending a link to each web resource weekly through an automated texting system.<br><br>Review - After an agreed period, contact the family to ask if they wish to meet with the practitioner to choose a new goal, troubleshoot any issues with their chosen goal or check on their child's progress.<br><br>○ Discuss their preferred method for being reminded to try this every day – e.g. using a paper diary, a reminder on their phone, a text message from an automated texting system etc.<br>○ Discuss their preferred option for reflecting regularly on how things are going – e.g. using a paper diary, making audio recorded notes on their phone, texting their HV team.<br>○ Engage with modelling, review and reflection activities – e.g. encourage families to look on the 'Tiny Happy People' website for example videos of their chosen goals and to note what the parent/caregiver on those videos did well	

	to support their child and/or to think of other things they could have done to increase their responsiveness.<br>○ Engage with motivational materials – provide a list of web resources which the parent/caregiver can explore which provide motivational information about why responsive interaction is so important and modelling that it can be a fun way to connect with their child. If this feels like too much information for the family, consider sending a link to each web resource weekly through an automated texting system.<br>○ The family then independently follow the programme over 2 – 3 months.<br>○ Review - after an agreed period contact the family to ask if they wish to meet with the practitioner to choose a new goal, troubleshoot any issues with their chosen goal or check on their child's progress.<br><br>**Level 3. A 'coaching' approach – with additional practitioner support**<br>The practitioner helps the parent/caregiver to record their goal and agree a schedule for visits to work together on this goal.	

	At subsequent visits,<br><br>• the practitioner watches a video with parent/caregiver showing families trying the chosen responsive behaviours in the chosen together time<br>• Practitioner models the behaviour with the child – e.g. "shall we try out getting down to his level? I will go first – let's play with his favourite toys and I am going to see if I can get down to his level"<br>• Encourage the parent to join in the play if they feel comfortable.<br>• Ask them to reflect on whether they think the chosen behaviour had an effect on how their child interacted<br>• If the parent/caregiver was confident enough to have a try ask them how that felt<br>• Set a goal for the following week and use a paper diary to record it and set reminders<br><br>Repeat the above weekly until the parent/caregiver is confident they are integrating the behaviour in their daily routines	

	Review - Practitioner judges whether to continue coaching with a new responsive interaction goal or suggest parent/caregiver chooses a new goal and works on it independently (i.e. moves to Level 2).	
**7. Optional additional support**	If, at step 3, the practitioner identifies additional barriers with respect to physical and social opportunities then additional support packages are offered.<br><br>Support package 1. access to early years setting/ social support Practitioner provides advice and guidance on accessing ECEC funding and placements. Barriers such as transport links or confidence to attend are problem-solved with the practitioner.<br><br>Locally designed signposting is provided identifying available local resources and settings.<br><br>Support package 2: access to age-appropriate books and play materials<br><br>Practitioner discusses ways to use everyday materials available at home to develop play	Locally designed signposting resources to available ECEC settings and parent/toddler sessions.<br><br>Locally designed signposting resources to

	and language and provides family with leaflet and links to resources on ‘Tiny Happy People’ and ‘’Hungry Little Minds’ webpages.	available book and toy lending libraries<br>Leaflets about how to use everyday materials available at home to develop play and language with QR code/web address links to ‘Tiny Happy People’ and ’Hungry Little Minds webpages’.
